# Addition of the microchromosome GGA25 to the chicken genome sequence assembly through radiation hybrid and genetic mapping

**DOI:** 10.1186/1471-2164-9-129

**Published:** 2008-03-17

**Authors:** Marine Douaud, Katia Fève, Marie Gerus, Valérie Fillon, Suzanne Bardes, David Gourichon, Deborah A Dawson, Olivier Hanotte, Terry Burke, Florence Vignoles, Mireille Morisson, Michèle Tixier-Boichard, Alain Vignal, Frédérique Pitel

**Affiliations:** 1 UMR INRA/ENVT Laboratoire de Génétique Cellulaire, INRA, Castanet-Tolosan, 31326, France; 2 Unité Expérimentale de Génétique Avicole, INRA, Nouzilly 37380, France; 3 Department of Biology, University of Leicester, Leicester, LE1 7RH, UK; 4 UMR INRA/INA PG Génétique et Diversité Animales, INRA, Jouy-en-Josas, 78352, France; 5 International Livestock Research Institute, PO Box 30709, 00100 Nairobi, Kenya; 6 Department of Animal & Plant Sciences, University of Sheffield, Sheffield, S10 2TN, UK

## Abstract

**Background:**

The publication of the first draft chicken sequence assembly became available in 2004 and was updated in 2006. However, this does not constitute a definitive and complete sequence of the chicken genome, since the microchromosomes are notably under-represented. In an effort to develop maps for the microchromosomes absent from the chicken genome assembly, we developed radiation hybrid (RH) and genetic maps with markers isolated from sequence currently assigned to "chromosome Unknown" (chrUn). The chrUn is composed of sequence contigs not assigned to named chromosomes. To identify and map sequence belonging to the microchromosomes we used a comparative mapping strategy, and we focused on the small linkage group E26C13.

**Results:**

In total, 139 markers were analysed with the chickRH6 panel, of which 120 were effectively assigned to the E26C13 linkage group, the remainder mapping elsewhere in the genome. The final RH map is composed of 22 framework markers extending over a 245.6 cR distance. A corresponding genetic map was developed, whose length is 103 cM in the East Lansing reference population. The E26C13 group was assigned to GGA25 (*Gallus gallus *chromosome 25) by FISH (fluorescence *in situ *hybridisation) mapping.

**Conclusion:**

The high-resolution RH framework map obtained here covers the entire chicken chromosome 25 and reveals the existence of a high number of intrachromosomal rearrangements when compared to the human genome. The strategy used here for the characterization of GGA25 could be used to improve knowledge on the other uncharacterized small, yet gene-rich microchromosomes.

## Background

The chicken is the first bird for which the complete genome was sequenced [1], providing a new viewpoint on the evolution of vertebrate genomes and useful data for the annotation of the human genome by the detection of conserved sequences [2,3]. The chicken karyotype is typical of birds, with the presence of chromosomes of extreme differences in size, comprising 38 pairs of autosomes – 5 macrochromosomes (GGA 1–5), 5 intermediate-sized chromosomes (GGA 6–10) and 28 microchromosomes – plus the Z and W sex chromosomes. Due to their very small size, microchromosomes cannot be identified by conventional cytogenetic techniques and require molecular labels, either BAC clones or chromosome paints, for identification by FISH (fluorescence *in situ *hybridisation) [4,5]. As a consequence, despite the good overall quality of the chicken draft assembly, which benefited from the relatively small content of repeat sequences, a large fraction corresponding to 10 of the smallest microchromosomes was missing in the first version of the published sequence assembly (February 2004) [6].

Alongside the sequence contigs attributed to the numbered chromosomes identified on the cytogenetic map, some contigs were linked to four small linkage groups of the genetic map that have not yet been assigned to chromosomes and that were therefore suspected of belonging to microchromosomes yet to be identified. However, these linkage groups contain only very limited amounts of sequence, ranging from 231 kb (E26C13), down to 1 kb (E64) [7]. Finally, around 120 Mb of sequence (165 Mb, when including gaps) were in the chrUn fraction, which contains any contig sequence that was not assigned to a chromosome or a linkage group from the genetic map [7]. Although a few supercontigs of the chrUn were large, with eight greater than 500 kb each, none covered more than 1.1 Mb and most (70%) were smaller than 2 kb [7]. The sizes of the smallest of the microchromosomes are difficult to estimate and various estimates, ranging from 3.4 [8] to 7 Mb [9] have been given. It appears therefore that a substantial fraction of the chicken genome is absent from the present sequence assembly, which could explain the fact that a number of genes present in mammals appear to be absent in the current chicken sequence assembly [1].

As a first step towards an improved sequence of the microchromosomes missing in the genome assembly, we started to build RH and genetic maps using markers developed from supercontigs selected from the chrUn. To guide the choice of the supercontigs, three criteria were used. First, under the hypothesis that a substantial amount of sequence exists in the ChrUn for the missing microchromosomes and that these microchromosomes could correspond to regions of human chromosomes of substantial size by conservation of synteny, we selected supercontigs from the chrUn that cluster in specific regions of human chromosomes by comparative genomics. Second, as microchromosomes tend to have a high G+C content, the supercontigs were chosen likewise. The last criterion was to only consider supercontigs larger than 10 kb. Amongst the several regions of human chromosomes meeting the three criteria, one on HSA1 (*Homo sapiens *chromosome 1) between positions 145–160 Mb was selected to develop an RH and genetic map. A BAC library was screened for markers suitable for FISH mapping that could then be used to identify the microchromosome covered by our new map.

## Results

### Selection of chicken supercontigs for the development of markers

**Figure 1:**
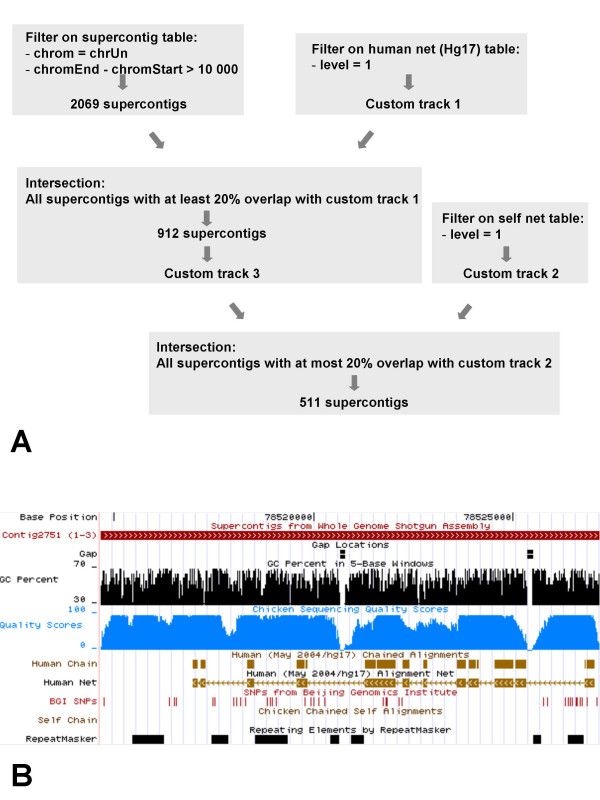
**Selection of supercontigs from chromosome Unknown (chrUn)**. The supercontigs from the chrUn are chosen with a minimal size of 10 kb, a minimal overlap with the human alignment net track of 20% and a maximum overlap with the chicken self-alignment table of 20%. A: schematic representation of the filtering and intersection queries in the UCSC table browser. B: screenshot of the UCSC browser, representing a supercontig selected with the above criteria. The overlap with the human alignment net track is about 75% and there is no overlap with another chicken region. The quality scores and repeat element tracks are displayed to guide the choice of PCR primers.

The chrUn fraction of the February 2004 assembly of the chicken genome is composed of 41,241 supercontigs, whose sizes vary between less than 1 kb, up to 1.1 Mb. Therefore, to work with a reasonable number of markers, we selected the 2069 supercontigs whose sizes were larger than 10 kb. These were selected using the filter in the table browser (Figure 1A). To obtain reliable indications on alignments of the chicken supercontigs in the human sequence, an intersection query was performed, requiring at least 20% overlap between the supercontigs and alignment net. Only level 1 net chains, which are the largest and highest scoring, were considered. Nine hundred and twelve supercontigs were thus selected. Finally, we wanted to avoid working with supercontigs from the chrUn that had a high similarity to known chicken chromosomes, as these could be due either to sequence fragments attributed to the chrUn following assembly artefacts, or to duplicated segments of the chicken genome that could have confounded the mapping through the amplification of two or more loci. Therefore, only supercontigs with a maximum of 20% self-overlap to the chicken sequence assembly, with level 1 alignment net, were retained. This resulted in a selection of 511 supercontigs (Figure 1A).

**Figure 2:**
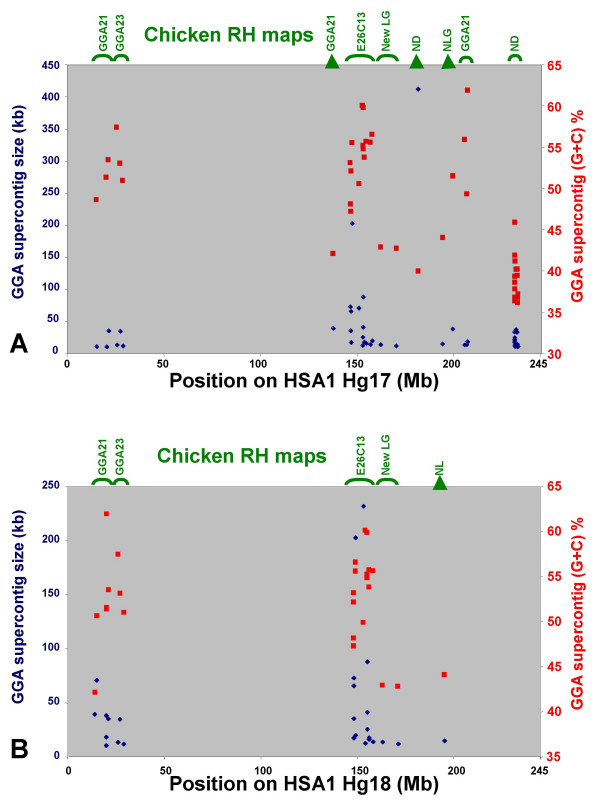
**Supercontigs selected on the basis of their position on HSA1**. The graphs represent the length (blue) and % (G+C) (red) of the chicken supercontigs and their position on the HSA1 chromosome assembly. In green: RH linkage group assignment of the supercontigs; LG: linkage group, ND: not done, NLG: new linkage group. A: position of all chicken supercontigs larger than 10 kb on HSA1 in the May 2004 Hg17 assembly. Supercontigs with a low % (G+C) were not selected for genotyping. B: position of the supercontigs genotyped in the RH panel on HSA1 in the March 2006 Hg18 assembly.

The position of the human alignment net of the chicken supercontigs in the human genome was determined using the UCSC database and plots representing these positions were inspected to determine the regions in which they clustered. An example for HSA1 is included in Figure 2. Regions of human chromosomes in which chicken supercontigs from the chrUn tend to cluster are good candidates for regions of conserved synteny between the human and chicken genomes that were missed in the chicken assembly. As an additional criterion suggesting that supercontigs belonged to microchromosomes, we selected supercontigs with a high G+C content, since the G+C content is higher in microchromosomes than macrochromosomes [1] (Figure 2).

Finally, information from the UCSC browser was used to guide the development of markers, by avoiding regions in which the chicken sequence was of low quality, where it contained repeat elements or where it was 100% similar to the human sequence (Figure 1B). This last criterion was used to avoid the amplification of the hamster DNA in the RH clones.

As a test case for our strategy of marker selection, we chose to study 26 chicken supercontigs with high G+C content that were clustered in three regions of HSA1 (Figure 2). When inspecting human chromosome 1 in the UCSC browser in the three selected regions, a similarity to sequence from the chicken E26C13 linkage group was found in the chicken alignment chain and alignment net tracks around position 147.5 Mb of HSA1. E26C13 is composed of sequence linked only to a linkage group of the genetic map [10]. As position 147.5 Mb of HSA1 is within the region in which most of the chicken chrUn supercontigs clustered, we decided to also develop markers for E26C13.

### Assignment of supercontigs to RH linkage groups

The markers developed for the 26 supercontigs and E26C13 were genotyped by PCR on the ChickRH panel [11], the results were submitted to the ChickRH server [12] and linkage groups were determined using the Carthagene software [13]. Two markers failed to produce satisfactory results, 7 markers corresponding to two regions of HSA1 mapped to the GGA21 linkage group, 3 markers to GGA23, 4 markers showed no linkage to a known chromosome or to a large linkage group and, finally, 13 markers were grouped into a new linkage group, together with 13 other markers from the ChickRH server database (Figure 2A). During the course of this work, the human sequence assembly was updated and the position of the supercontigs mapped in chicken by RH mapping was re-evaluated. As a result, the markers of GGA21 that were dispersed in the Human May 2004 sequence assembly Hg17 (Figure 2A) are now grouped together in the Human March 2006 sequence assembly Hg18 (Figure 2B).

All the 13 markers of the new large linkage group, including the one from E26C13, were developed from chicken supercontigs with sequence similarity to the region 147.7 to 158 Mb on HSA1 in the Hg17 assembly and are now in the region 148 to 158 Mb in Hg18 (Figure 2). This 10-Mb-long region of chromosome 1 seems thus to present conservation of synteny with a segment of the chicken genome that corresponds to the E26C13 linkage group of the genetic map, which was not linked to the karyotype. A first RH framework map built with 7 of the 26 markers of the E26C13 linkage group spanned 188 centiRays (cR) (data not shown).

### BAC screening and assignment of E26C13 to GGA25 by FISH mapping

The Wageningen BAC library, which has a 5.5-fold coverage of the genome [14], was screened by PCR with 4 markers from the E26C13 RH linkage group: two ESTs (Expressed Sequence Tags), *ARHGEF11 *and *COPA*, from the middle of the map and two STSs (Sequence Tagged Sites), *SEQ1010 *and *SEQ1021*, from each end. Four clones: bw64M19, bw64M20, bw88F2, bw90F5, were obtained with *ARHGEF11 *and *COPA *and no clones could be detected with the two other markers (Table 1).

**Table 1:** BAC screening results

**RH marker**	**positive clone**
100A3M13	bw25B06, bw27G08, bw93G01
ARHGEF11	bw64M19, bw64M20
COPA	bw88F2, bw90F5
GCT1888	none
GCT1893	none
GCT1967	none
SEQ0426	bw83P09
SEQ1010	none
SEQ1021	none
SEQ1285	none
SNP105	bw22L24, bw64M19
SNP115	none
SNP123	none
SNP18	bw83P09
SNP29	none
SNP36	none
SNP42	none
SNP46	none
SNP50	none
SNP54	bw83P09
SNP95	bw120G10

**Figure 3:**
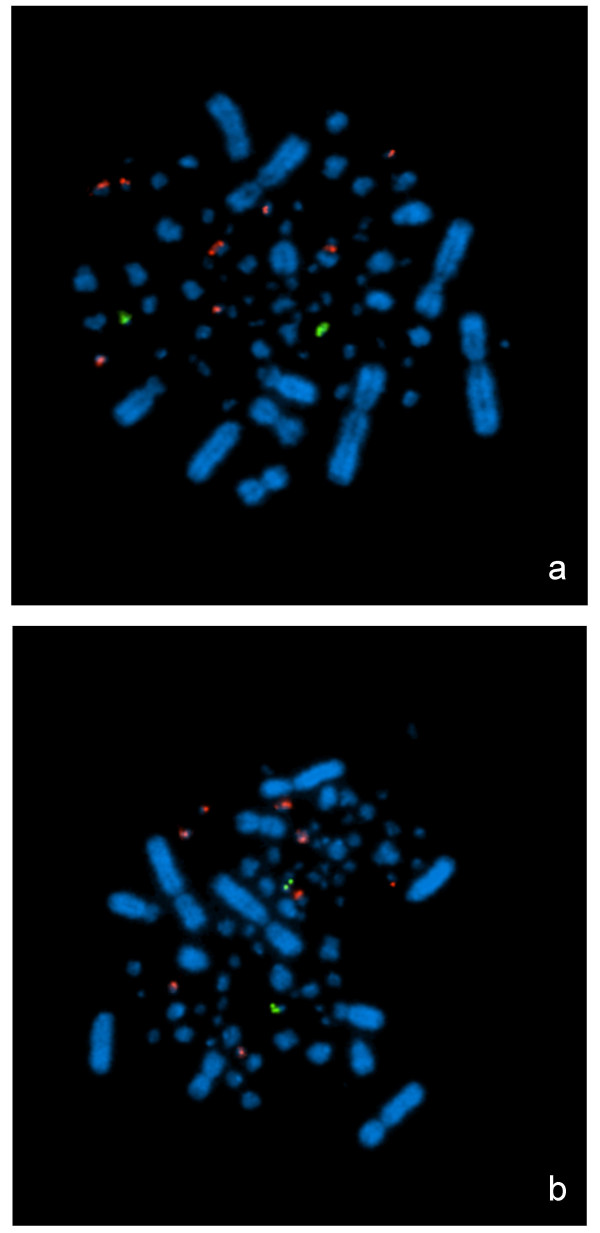
**FISH assignment of E26C13 to GGA25**. Dual-color FISH was used to identify the microchromosome corresponding to E26C13. Eight of the BAC clones known to tag microchromosomes of similar size as the one hybridised by the BAC clone from E26C13 are shown: (a): BAC clones for GGA19, GGA21, GGA22, GGA23 (red) and BAC clone bw90F5 for E26C13 (green). (b): BAC clones for GGA24, GGA26, GGA27, GGA28 (red) and BAC clone bw90F5 for E26C13 (green).

All four BAC clones co-localised by two-colour FISH to the same microchromosome, whose size could be estimated to be between chromosomes 25 and 27. To identify the microchromosome to which the E26C13 linkage group corresponds, the BAC bw90F5, that gave the strongest hybridisation signal, was selected for hybridisation by two-colour FISH with the clones that tag the microchromosome pairs of similar size, as described in Masabanda et al [5]. To ensure a good coverage of microchromosomes in the size range of the one identified by bw90F5, clone tags were used for microchromosomes GGA19 to GGA32, with the exception of GGA25, for which no clone had yet been identified by the chicken cytogenetic community. For all combinations of tag clones used with bw90F5, distinct signals on two different chromosome pairs were obtained (Figure 3), indicating that the E26C13 linkage group corresponds to GGA25, which was missing in the clone tag collection and by extension in the sequence assembly of the genome.

Sequence and position information on the 26 markers of the first GGA25 RH map were sent to Wes Warren and LaDeana Hiller (Washington University, Saint Louis, MO) who integrated these data into the second assembly of the chicken genome, which was released in May 2006. This allowed the first attribution of sequence data to GGA25. The sequence assembly of GGA25 in the second chicken genome build contains 1.36 Mb of attributed sequence and the coverage is just over 2 Mb when including gaps. Since the extension of the RH map (see later), seven additional BAC clones were obtained by screening the library with 17 other GGA25 markers (Table 1).

### Adding markers to the GGA25 RH map

**Figure 4:**
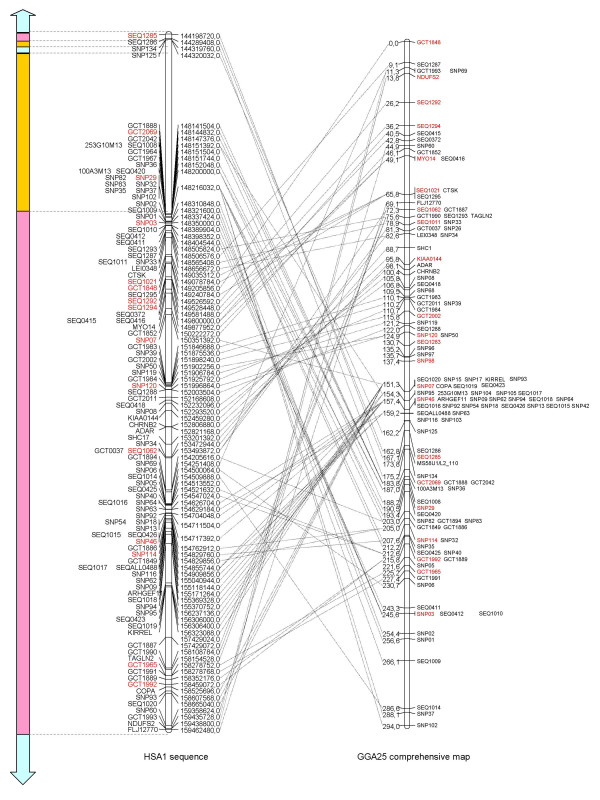
**Comparison between RH map and human genome assembly**. The RH map (right) obtained in this study is compared to the human chromosome 1 (middle) sequence assembly (Hg18) in the region 144.1–159.5 Mb [37]. For each marker on the framework map, a line joins both positions (cR and Mb) together. Framework markers are in red. Left: conservation of synteny between HSA1 and chicken chromosomes. Pink: GGA25, blue: GGA08, orange: GGA01.

Since we have now defined a region of conserved synteny between GGA25 and HSA1, additional markers were developed using this information. One hundred and five markers were developed from additional chrUn contigs of shorter length than the ones selected for building the preliminary map. These were selected in the chicken 2004 assembly with the UCSC browser using the criterion of an alignment with the region in HSA1 between 144–160 Mb. Thirty-four markers were developed from chicken EST sequences orthologous to human genes from the same region, obtained from the NCBI [15] or SIGENAE [16] databases. Interestingly, 16 of these EST contigs did not present any similarity to the chicken genome assembly, suggesting they were missed in the whole genome sequencing process. Two markers were developed from the GGA25 assembly when it became available in March 2006, BAC sequencing provided two markers and one marker was obtained from a charomid clone containing minisatellite marker LEI0013 present in the E36C13 genetic map [see Additional file 1]. Altogether, 112 primer pairs out of 143 (78%) enabled successful amplification and the subsequent mapping of the corresponding fragments: 94 to GGA25 and 18 to other chromosomes. With the addition of those new markers, the total number of markers now assigned to GGA25 is 120. After multipoint analysis, the framework map comprised a total of 22 markers for a length of 245.6 cR The remaining 98 markers were integrated at their best possible locations, to build a comprehensive map (Figure 4). These new results allowed us to assign 25 additional contigs from the 2006 chrUn assembly to GGA25 [see Additional file 1].

### Genetic markers and genetic map

**Figure 5:**
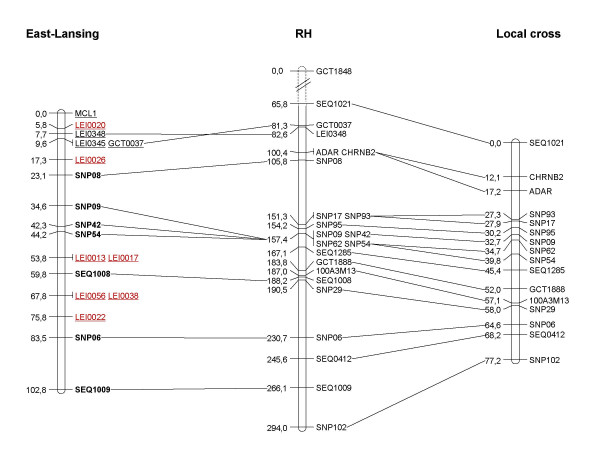
**Radiation hybrid (RH) map/genetic map comparison for chicken chromosome 25**. The RH map (in cR) is compared to the genetic map (in cM) obtained from the East Lansing chicken reference backcross mapping population (left) and our experimental cross (right). Underlined: international markers [10], red: minisatellite markers, bold: new markers (this study).

Sequence information from the markers on the GGA25 RH map was used to develop genetic markers to build genetic maps. Two maps were built: one based on one of our experimental populations and the other by using the East Lansing [17] chicken reference backcross mapping population, so as to integrate markers mapped by others. The markers informative in our experimental population enabled us to build a genetic map 77 cM long, comprising 16 framework markers (Figure 5). Seven informative markers were added to the E26C13 linkage group in the East Lansing genetic map. Together with the data already available, E26C13-GGA25 is now composed of 18 markers and is 103 cM long (Figure 5).

## Discussion

We used a strategy based on the comparative mapping of sequence contigs from the chrUn fraction of the chicken genome and from chicken ESTs to characterize a chicken microchromosome that was absent from the first sequence assembly. Isolation of BAC clones and FISH mapping enabled us to identify this microchromosome as being GGA25. Although GGA25 is now included in the second version (May 2006) of the chicken genome assembly, our RH maps enabled us to assign 23 additional contigs from the chrUn, amounting to a total of 44.8 kb of sequence, and 18 ESTs that did not have any significant BLAST hit. Genetic maps of GGA25 were successfully developed by using the data from the RH maps.

The difficulty we encountered in isolating BAC clones and the small size of many of the chrUn contigs we attributed to GGA25 suggest that there may be a cloning and/or a sequencing bias against obtaining sequence from microchromosomes. These difficulties in obtaining sequence have since been confirmed for other microchromosomes by us and others (J. Dodgson, personal communication). The GGA25 sequence in the May 2006 assembly has a composition of 52.4% G+C, which is amongst the highest for chicken chromosomes [1]. The chrUn contigs that we have added to GGA25 through RH mapping have a mean G+C content of 61.2% [see Additional file 1]. This confirms GGA25 to be amongst the most GC-rich microchromosomes. The observation from FISH experiments [5], and the high proportion of minisatellite markers on the E26C13 genetic map [10], suggest that GGA25 contains a high number of repetitive sequences. Both the high G+C content and a high proportion of repetitive elements may account for the paucity of available sequence for this chromosome in the chicken genome assembly at the beginning of this study.

An average retention frequency of 24.1% (range 11.2–39.3%) was observed for the 120 GGA25 markers studied here. This finding is in agreement with previously observed values [18,19], confirming a higher retention rate for micro – than for macrochromosomes.

By using the average value of 38.7 kb/cR observed for other chromosomes mapped with the ChickRH6 panel [19], the length of GGA25 can be estimated to be around 11.4 Mb, which is in accordance with the expected size for this chromosome [9]. This suggests also that the entire chromosome is covered by our RH map. The portion of human chromosome 1 orthologous to GGA25, located at HSA01q21.2-q23.3, is about 11.5 Mb long (from 148.1 to 159.5 Mb, in addition to a short fragment of about 100 kb around 144.2 Mb, Hg18). This region corresponds to a chicken microchromosome covering about 11–12 Mb, giving a ratio of about 1 Mb HSA/1 Mb GGA, which is much lower than the average ratio when considering the whole genome (2.4 Mb HSA/1.0 Mb GGA), and may be associated with the very high density of genes in this portion of the human genome [20,21].

The HSA1 region orthologous to GGA25 contains around 410 genes [20]. Assuming most of these genes are present in GGA25, the gene density of this microchromosome would be 5.9 genes/100 kb, which is comparable to the value of 5.5 obtained for GGA32, and higher than the ratio observed for longer chicken chromosomes, as expected [22].

The RH results indicate a high number of chromosomal rearrangements in the chicken and human lineages in the region corresponding to GGA25 (Figure 4). The high number of intra-chromosomal rearrangements within the region of conserved synteny between birds and mammals is in accordance with results obtained for other chromosomes, e.g., GGA02 [23], GGA05 [24], GGA07 [18], GGA10 [25], GGA14 [26], GGA15 [27] or GGA28 [28].

Genotyping the East Lansing population allowed us to connect our results to the international chicken reference backcross mapping pedigree, and to develop a single-locus marker from a minisatellite previously mapped in this population. These results suggest that minisatellite-type sequences are distributed throughout GGA25 (Figure 5).

The genetic map constructed using our local cross presents smaller genetic distances between markers than the East Lansing map. The variation observed are most likely the result of differences between the lines used for the two crosses. By using the genetic map built with our local cross, we find an overall ratio of 3.0 cR/cM, which is a relatively low value when compared to the ratio obtained for chicken macrochromosomes [18,23,24], but close to the ratio of 3.6 obtained for chromosome 14 [26]. This result is in accordance with the fact that the recombination rate is negatively correlated with the physical length of the chromosome [1].

## Conclusion

The availability of more than 2.5 million single-nucleotide polymorphisms (SNP), and the improvement of genotyping methods, will lead to very dense genetic and RH maps in chicken. However, most of these SNPs are situated at known chromosomal locations in the chicken genome and a large effort is still needed to identify several microchromosomes which are still absent from the chicken genome map. The integrated strategy used here, by using comparative mapping and the sequence data currently assigned to the chrUn as resources, along with using FISH, RH and genetic mapping as complementary tools for the characterization of GGA25, may be a way to improve our knowledge of the gene-rich microchromosomes that as yet remain uncharacterized. Strategies that include microdissection, flow sorting or magnetic-bead chromosome isolation may however be necessary to achieve this goal.

## Methods

### Initial selection of chicken supercontigs

The selection of supercontigs in the chicken genome assembly was done by performing queries in the table browser of the Chicken (*Gallus gallus*) Genome Browser Gateway at UCSC [7]. At the time when the queries were performed, the chicken assembly available was the February 2004 version and the human alignment net track displayed was a comparison to the Hg17 assembly. Queries on the chicken supercontigs were intersected with queries on the human genome net (the best human-chicken alignment for every part of the chicken genome) and chicken self-chain (alignment of the chicken genome with itself) tracks. For each supercontig, information provided by the UCSC browser was used to select a region for primer design by avoiding repeat elements, and to avoid sequence with high similarity to the human genome sequence and poor-quality sequence.

### Selection of additional RH markers

Several sources of markers putatively mapped to E26C13 were used: the human genome sequence, chicken chrUn sequence, chicken BAC clones and one chicken charomid clone containing a minisatellite.

Human genes from regions for which available comparative mapping data suggested a conservation of synteny with E26C13 were selected for marker development. Chicken EST sequence alignments to these human genes were visualised by using the ICCARE software [29], to guide the designing of primers.

Additional chicken chrUn contigs showing alignment with the human region of interest were selected from the Chicken (*Gallus gallus*) Genome Browser Gateway at UCSC [7], first from the Feb. 2004 assembly, and subsequently from the May 2006 version when it became available.

BAC clones from the genomic region of interest were obtained by screening the Wageningen BAC library [14] with markers selected from the E26C13 RH map. Primers were designed after end sequencing.

A charomid library had been previously constructed using the same method as in [30] and a clone (MSL58) isolated containing LEI0013, a chicken minisatellite which mapped to E26C13 (Hanotte et al., unpublished data; [10]). A first sequence fragment was obtained from the charomid DNA with vector-specific primers U (5'-CGTATCACGAGGCCCTTTC-3') and L (5'-TGACAGCTTGTATGTTTCTGC-3'). This sequence was then extended by using an internal primer (5'-CCCTCCTCTTGTGTTTAATTA-3'). DNA extraction and sequencing reactions were performed as for the BAC clones (see below).

Primer data, accession numbers and other information on the sequences used for marker development are given in Additional file 1.

### Primer design

Primers for RH mapping or BAC screening were chosen by using the Primer3 server [31].

### BAC screening

The BAC clones were selected in the Wageningen library by two-dimensional PCR screening of superpools and pools arranged in microplates as described in [14]. PCR amplifications were carried out for each marker in 25-μl reactions containing 25 ng DNA, 0.4 μM primers, 0.25 units Taq polymerase (Life Technologies-GIBCO BRL), 2 mM MgCl_2 _and 0.2 mM dNTP on a GeneAmp PCR System 9700 thermocycler (Applied Biosystems). The first 5-min denaturation was followed by 36 cycles, each of denaturation at 94°C for 30 s, annealing at marker T_m _for 30 s and elongation at 72°C for 30 s. PCR products were analysed on 2% agarose gels, electrophoresed in 1 × TBE buffer, and visualized by ethidium bromide staining. DNA was extracted from BAC clones by alkaline lysis according to Qiagen procedures (midi kit, Qiagen).

### BAC end sequencing

Sequence reactions were performed for both ends using the diChloroRhodamine Prism AmpliTaq FS Big Dye Terminator V3.1 kit (Applied Biosystems) and M13 forward or M13 reverse sequence primer. Sequences were analysed on an ABI 3730 sequencer (Applied Biosystems). Sequences providing new information were selected for marker development.

### Fluorescence *in situ *hybridisation

FISH was carried out on metaphase spreads obtained from embryo fibroblast cultures arrested with 0.06 μg/ml colcemid and fixed by standard procedures. Two-colour FISH was performed by labelling 100 ng of each BAC with digoxigenin-11-dUTP (Roche Diagnostics) or biotin-16-dUTP (Roche Diagnostics) by random priming. Probes labelled with biotin and with digoxigenin were ethanol precipitated together and hybridised to the metaphases for 24 hours at 37°C in a Dakomation hybridizer after denaturation at 72°C for 8 min. Biotin-labelled probes were detected with Alexa 594 antibodies (Invitrogen) and digoxigenin-labelled ones with Alexa 488 antibodies (Invitrogen). Chromosomes were counterstained with DAPI (4', 6-diamidino-2-phenilindole-dihydrochloride) in Vectashield antifade solution (Clinisciences). Two-colour FISH was performed either with one probe labelled in each colour or with groups of two or three clones for each colour. The hybridised metaphases were screened with a Zeiss fluorescence microscope and a minimum of twenty spreads was analysed for each experiment. Spot-bearing metaphases were captured and analysed with a cooled CCD camera using Cytovision software (Applied Imaging).

### Radiation hybrid mapping

#### PCR amplification

The generation of the RH panel has already been described [11].

PCR amplifications were carried out for each marker in 15-μl reactions containing 25 ng DNA, 0.4 μM of each primer, 0.25 units of Taq polymerase (Life Technologies-GIBCO BRL), 2 mM MgCl_2_, 0.2 mM dNTP on a GeneAmp PCR System 9700 thermocycler (Applied Biosystems). The first 5-min denaturation was followed by 36 cycles, each consisting of denaturation at 94°C for 30 s, annealing at T_m _for 30 s and elongation at 72°C for 30 s. PCR products were analysed on 2% agarose gels, electrophoresed in 1 × TBE buffer, and visualized by staining with ethidium bromide.

Each marker was genotyped twice and a third genotyping was performed in cases of discrepancy between the first two experiments.

#### RH map construction

The genotyping data obtained were analysed with the Carthagene software [13]. A group of markers for E26C13 was defined by two-point analysis using a LOD threshold of 8. By using all the markers from this group, a 1000:1 framework map (a map whose likelihood is at least 1000-fold higher than the next possible highest likelihood using the same markers in alternate orders) was built under a haploid model. This framework was constructed using a stepwise locus-adding strategy, starting from the triplet of markers whose order is the most likely ("buildfw" option). The framework map thus automatically built was further improved towards larger distance coverage by removing markers that prevented its extension. The different provisional framework maps were checked by using a simulated annealing greedy algorithm, testing for possible improvements of the map by inversion of large fragments, and a flips algorithm testing all possible local permutations within a sliding window of six markers. After validation of the framework map built under the haploid model, the distances between the framework markers were re-evaluated under a diploid model. Finally, markers not included in the framework map were mapped relative to it, to determine their most likely positions.

All maps were drawn with MapChart 2.0 [32].

### Genetic mapping

PCR amplifications were performed as for RH mapping, except for the occasional use of fluorescent primers. Genotyping was performed through SSCP (Single-Strand Conformation Polymorphism) and silver staining, [33,34], except for SNP95, SEQ1285 and 100A3M13, which were analysed by fluorescent SSCP on an ABI 3100 sequencer (Applied Biosystems), as described in Applied Biosystems Publication 116AP01-02. GCT1888 was genotyped through a PCR-RFLP analysis with the restriction enzyme *Tse*I (New England Biolabs). The microsatellite marker SEQ0412 was analysed on an ABI 3730 sequencer (Applied Biosystems).

Segregation analyses were performed in the East Lansing chicken reference back-cross mapping population [17], and genetic localizations on the maps calculated with the Mapmanager software [35]. An experimental population derived from a Fayoumi ancestor, consisting of two chicken half-sib families (Sire1 × 5 females, 115 offspring; Sire2 × 6 females, 94 offspring) was also used to build a second genetic map. For this population, linkage analysis was performed using CriMap version 2.4 software [36]. The build option was used to order markers within the linkage group. The flips option was used to examine the order of the different loci by inverting every two or three loci.

## Authors' contributions

MD, KF, MG, SB, FV and FP carried out the molecular studies. VF completed the FISH experiments. DG and MTB provided the local pedigree. MM made the RH panel and the very first RH map of E26C13. The minisatellite markers for E26C13 were developed by DD, OH and TB. FP built the final maps. FP and AV conceived the study, and participated in its design and coordination. FP, VF and AV drafted the manuscript. DD, AV and TB finalised the manuscript. All authors read and approved the manuscript.

## Supplementary Material

Additional file 1 Markers mapped on the GGA25 chicken comprehensive radiation hybrid map. The data provided represent the detailed description of the 120 markers mapped on the GGA25 chicken comprehensive radiation hybrid map. Click here for file
